# Percutaneous calcium sulfate injection versus localized scrape bone grafting: clinical effect comparison in titanium elastic nail treatment of pathological fracture of proximal humerus caused by unicameral bone cysts in children

**DOI:** 10.3389/fped.2023.1334950

**Published:** 2024-01-09

**Authors:** Yuyin Xie, Zhenqi Song, Zhongwen Tang, Zheng Xu, Zhouzhou Rao, Jie Wen, Sheng Xiao

**Affiliations:** ^1^ Department of Pediatric Orthopedics, Hunan Provincial People’s Hospital, the First Affiliated Hospital of Hunan Normal University, Changsha, Hunan, China; ^2^ Key Laboratory of Translational Cancer Stem Cell Research, Department of Physiology, Hunan Normal University School of Medicine, Changsha, Hunan, China; ^3^ Department of Anatomy, Hunan Normal University School of Medicine, Changsha, Hunan, China

**Keywords:** unicameral bone cyst, titanium elastic nails, percutaneous calcium sulfate injection, localized scrape bone grafting, pathologic fracture

## Abstract

**Objective:**

A retrospective study was conducted to compare the mid-term clinical efficacy between percutaneous calcium sulfate injection (PCSI) and localized scrape bone grafting (LSBG) in using titanium elastic nails treat humerus pathologic fractures caused by unicameral bone cysts in children.

**Methods:**

Humerus pathologic fracture patients with unicameral bone cysts in our pediatric orthopedic department from January 2015 to January 2020 were retrospectively analyzed. Patients were divided into two groups, namely the PCSI group and the LSBG group, based on the type of bone grafting material they received. Preoperative assessments were made in both groups using the Cyst Index and Cyst activity. During the perioperative phase, assessments were made regarding operative time, bleeding, postoperative VAS scores, and the frequency of reoperation within 2 years. Clinical outcomes were evaluated using the Capanna scale at the last follow-up, and the occurrence of re-fractures during the follow-up period.

**Results:**

A total of 22 patients were included, with a mean follow-up duration of 33.5 ± 5.8 months. No significant differences were found between the two groups in terms of Cyst Index and Cyst activity before the operation. The operative time, bleeding, and postoperative VAS scores in the PCSI group were found to be lower than those in the LSBG group (*P* < 0.05). The PCSI group also showed a higher frequency of reoperation within 2 years compared to the LSBG group (*P* < 0.05). However, no significant differences were observed between the two groups in terms of Capanna scale scores at the last follow-up and the incidence of re-fractures during follow-up.

**Conclusions:**

Both titanium elastic nails (TEN) combined with PCSI or LSBG were found to be safe and effective treatments for humerus pathologic fractures caused by unicameral bone cysts in children. PCSI is considered as a less invasive option with shorter operative times, less bleeding, and reduced postoperative pain, although it comes with the risk of multiple injections. On the other hand, LSBG is considered as a more invasive option for the treatment of active bone cysts but is associated with a lower recurrence rate.

## 1 Introduction

Unicameral bone cyst (UBC), first reported by Virchow in 1876, has gained gradual recognition (1). The most prevalent site for these lesions is the proximal humerus, followed by the proximal femur, and they are frequently associated with pathological fractures (2). Due to the unclear understanding of the disease's pathogenesis, the treatment of unicameral humeral bone cysts in children remains a topic of debate (3). This study collected clinical manifestations, pertinent imaging data, surgical techniques, and pathological examination reports from children with unicameral humeral bone cysts to analyze the effectiveness of titanium elastic nails fixation combined with minimally invasive artificial bone injection or titanium elastic nails fixation combined with debridement and allogeneic bone grafting open surgery.

## 2 Materials and methods

### 2.1 Study population

This study included patients with humerus pathologic fractures caused by unicameral bone cysts in our department from January 2015 to January 2020. All patients and their parents signed an informed consent form for the procedure.

Inclusion Criteria: (1) Age less than 14 years; (2) Confirmed diagnosis of pathologic fracture with biopsy results confirming unicameral bone cyst; (3) Surgical intervention involving titanium elastic intramedullary nail fixation combined with artificial bone injection and open treatment with titanium elastic intramedullary nail fixation combined with focal removal and allogenic bone graft transplantation; (4) Follow-up for a minimum of 24 months.

Exclusion Criteria: (1) Biopsy results confirming other conditions, such as aneurysmal bone cyst; (2) Loss of clinical data during follow-up.

### 2.2 Surgical procedures

#### 2.2.1 Titanium elastic intramedullary nail placement

All patients were placed in a supine position and administered general anesthesia. The affected limb was abducted, and standard disinfection and draping were performed. Small incisions were made 1–2 cm above the medial and lateral aspects of the humeral ankle, and these incisions were subsequently extended. A pre-curved flexible intramedullary nail, constructed from titanium, was inserted retrograde into the proximal humerus.

#### 2.2.2 Implantation of bone repair material

##### 2.2.2.1 Percutaneous injection of calcium sulfate

The patient was positioned in a supine manner on the operating table, with the affected limb placed on the lateral surgical table. Using fluoroscopy, the humeral lesion was located. Three 16-gauge bone marrow puncture needles were employed to access the lesion from various angles and directions. The fluid was aspirated from the cyst, and a tissue sample from the lesion was obtained for biopsy. The lesion was thoroughly irrigated with saline and complex iodine. A puncture needle was employed to scrape the bone trauma within the lesion until it became clear. Following the injection, the bone puncture needle was withdrawn, and a sterile dressing was applied.

##### 2.2.2.2 Focal removal of allograft bone grafting

The patient was positioned supine on the operating table, and the affected limb was placed on the lateral operating table. C-arm fluoroscopy was used to precisely locate the humeral lesion, and the surgical field was once again disinfected with alcohol. An incision was made through the skin, subcutaneous tissue, and deep fascia at the site of the lesion. The periosteum was carefully dissected, exposing the cortical portion of the lesion. A bone window was made by osteotome while avoiding harm to the epiphysis. The capsule wall was scraped as thoroughly as possible, and tissue from the lesion was obtained for biopsy. The bone cavity was filled with allograft bone in close alignment with the natural trabecular pattern of the bone. A portion of the windowed bone cortex was reimplanted into the window. After surgery, the affected limb was immobilized using an abductor brace.

### 2.3 Data collection

Patient assessments included preoperative imaging analysis based on the Cyst Index and Cyst activity. Perioperative evaluations encompassed operative duration, bleeding volume, postoperative VAS scores, and the frequency of reoperation within the first two years. Final clinical assessments during follow-up included the Capanna scale at the last follow-up and the incidence of refractures.

1. Cyst Index (4): The cyst area was determined by outlining the border using the area measurement tool located on the right-hand toolbar of the PACS system, and then calculated using the following formula $\left(\mathrm{Cyst}\;\mathrm{index}\;=\;\frac{\mathrm{Cystarea}}{\mathrm{diaphysis}\;\mathrm{diamete}r^{2}}\right)$
2. Cyst activity (5): A lesion situated less than 10 mm from the proximal epiphyseal plate of the humerus was categorized as an active cyst, while those greater than 10 mm were classified as latent cysts.
3. Postoperative pain: Post-operative pain was assessed using a visual analog scale (VAS) score on the third day post-operation, where a score of 0 mm indicated the absence of pain, and 100 mm indicated unbearable pain (6).
4. Capanna Bone Cyst Treatment Scale (7): This scale included the following categories: (i) cured: the cyst cavity was completely filled with bone without any residue; (ii) cured but with residue: most of the cyst cavity was filled with bone, there was fusion of the graft with the humerus, thickening of the bony cortical margin, but with a small residual translucent area; (iii) recurrence: initially cured, but subsequently a translucent area and thinning of the bony cortex appeared; (iv) no response to treatment: no evidence of any cure.

In the PCSI group, there were 12 patients (7.5 ± 1.7 years old, 6 males, 6 females) with one case (8%) of active bone cysts. The LSBG group comprised 10 patients (7.8 ± 1.1 years old, 6 males, 4 females) with one case (10%) of active bone cysts. Both groups were followed for a minimum of 24 months (mean follow-up of 33.5 ± 5.8 months), with the shortest follow-up being 25 months. No significant differences were observed between the two groups in terms of demographic parameters, including gender, age, and affected side. General information about the selected patients is provided in Table 1.

**Table 1:** General information of selected patients.

No	Gender	Age	L/R	FU	Group	Cyst index	Cyst activity	OP time	BL	VAS score	OT in 2Y	Capanna LF	Refracture times
1	F	6	L	26	PCSI	3.6	3.5	86	25	40	4	2	1
2	M	7	R	32	LSBG	2.9	4.1	130	60	70	1	2	0
3	M	7	R	31	PCSI	4.1	8.8	80	30	30	4	3	0
4	M	11	L	28	PCSI	4.3	6.3	95	35	50	3	2	1
5	F	6	R	37	PCSI	4.7	4.2	98	20	50	3	3	0
6	F	8	R	40	LSBG	3.1	3.8	145	65	80	2	3	1
7	M	9	L	29	LSBG	4.2	7.1	150	70	60	1	2	0
8	F	6	R	33	PCSI	2.8	5.3	100	40	40	4	2	0
9	M	7	R	28	PCSI	3.3	5.9	80	35	40	2	2	0
10	F	8	L	25	LSBG	2.1	3.9	142	60	70	1	2	0
11	M	10	L	37	LSBG	4.3	8.2	138	80	70	1	2	1
12	M	6	R	36	PCSI	3.9	2.4	88	30	30	3	2	1
13	M	7	L	29	LSBG	3.5	4.3	148	65	80	0	2	0
14	F	7	L	38	PCSI	2.9	1.2	105	50	40	4	3	1
15	F	9	R	44	LSBG	3.1	5.2	129	60	80	0	2	0
16	M	6	L	42	LSBG	3.5	0.4	155	80	90	1	2	0
17	F	10	L	41	PCSI	3.5	7.1	102	35	40	3	2	0
18	M	9	L	32	PCSI	4.1	0.9	90	25	30	3	3	0
19	M	7	L	30	LSBG	4.2	5.3	138	70	80	1	3	1
20	F	8	R	27	LSBG	3.9	5.8	148	75	70	1	2	1
21	M	8	L	31	PCSI	3.1	7.2	82	30	20	4	2	1
22	F	7	R	42	PCSI	2.3	6.9	93	30	40	2	2	0

### 2.4 Statistical analysis

Data were analyzed using SPSS 21.0. The collected data were presented as absolute numbers and percentages. Statistical analysis was conducted using Student's *t*-test to identify differences in treatment response between the two groups. A significance level of *p* < 0.05 was considered statistically significant.

## 3 Results

A total of 22 eligible patients were included in this study, with 12 patients (7.5 ± 1.7 years old, 6 males, 6 females) in the PCSI group and 10 patients (7.8 ± 1.1 years old, 6 males, 4 females) in the LSBG group.

**Figure 1:**
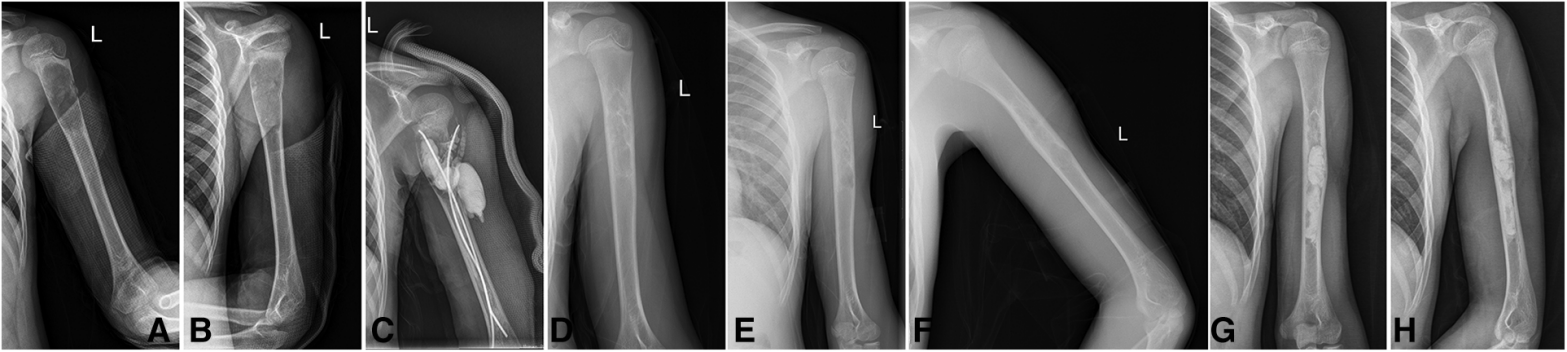
Typical case in PCSI group. Boy, age 9, Case No.18, Unicameral bone cysts of left humerus, Cyst index: 4.1, Cyst activity: 0.9, OP Time: 90 min, BL: 25 ml, VAS Score: 30, OT in 2Y: 3 times, Capanna LF: 3, Refracture Times: 0 time. (**A,B**) x-rays pre-operation; (**C**) x-rays post-operation, (**D**) x-rays after TEN removal, (**E,F**): recurrence in 12 months FU, (**G,H**) x-rays after second PCSI injection.

In the PCSI group, the average Cyst Index before the operation was 3.55, the average Cyst activity before the operation was 4.98, the average operative time was 91.6 min, the average intra-operative bleeding was 32.1 ml, the average postoperative VAS score was 38.3, the average operation frequency within 2 years after the first surgery was 3.25 times, the average Capanna scale at the last follow-up was 2.3, and the average re-fracture frequency was 0.4 times. A typical case is illustrated in Figure 1.

**Figure 2:**
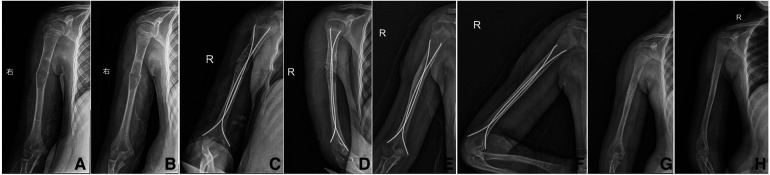
Typical case in LSBG group. Girl, age 9, Case No.15, Unicameral bone cysts of right humerus, Cyst index: 3.1, Cyst activity: 5.2, OP Time: 129 min, BL: 60 ml, VAS Score: 80, OT in 2Y: 0 times, Capanna LF: 2, Refracture Times: 0 time. (**A,B**) x-rays pre-operation; (**C,D**) x-rays post-operation, (**E,F**) No recurrence in 24 months FU, (**G,H**) x-rays after TEN removal.

In the LSBG group, the average Cyst Index before the operation was 3.48, the average Cyst activity before the operation was 4.81, the average operative time was 142.3 min, the average intra-operative bleeding was 68.5 ml, the average postoperative VAS score was 75.0, the average operation frequency within 2 years after the first surgery was 0.90 times, the average Capanna scale at the last follow-up was 2.2, and the average re-fracture frequency was 0.4 times. A typical case is illustrated in Figure 2.

As presented in Table 2, there were no significant differences between the two groups in terms of Cyst Index and Cyst activity before the operation. However, the PCSI group exhibited significantly shorter operative times, less intra-operative bleeding, and lower postoperative VAS scores compared to the LSBG group (*P* < 0.05). The PCSI group also had a significantly higher frequency of reoperation within 2 years after the initial surgery compared to the LSBG group (*P* < 0.05). However, there were no significant differences between the two groups in terms of Capanna scale scores at the last follow-up and the incidence of re-fractures during the follow-up period.

**Table 2:** Comparison of PCSI group and LSBG group.

	PCSI group	LSBG group
Total	12	10
Gender		
Male	6	6
Female	6	4
Side		
Left	6	6
Right	6	4
Age	7.5	7.8
FU	33.6	33.5
Pre OP		
Cyst index	3.55	3.48
Cyst activity	4.98	4.81
Peri- OP		
OP time	91.6	142.3^a^
BL	32.1	68.5^a^
Post- OP		
VAS	38.3	75.0^a^
OF	3.25	0.9^a^
Results		
Capanna scales	2.3	2.2
Re-fracture frequency	0.4	0.4

^a^ *P* < 0.05.

## 4 Discussion

Unicameral bone cysts are a common occurrence in children and adolescents, with a higher prevalence among males (8, 9). In most cases, patients are asymptomatic, experiencing only mild pain or pressure. In instances of pathological fractures, the bone within the cyst wall may present as small “ice-cracked” bone fragments, which can appear as if they are “trapped” within the cystic cavity (10).

The most widely accepted etiological theory suggests that the impaired intraosseous venous return is a key factor. Some researchers have investigated the role of oxygen radicals, osteoprotegerin (OPG), and nuclear factor-kB receptor activator ligand (RANKL) expression levels in the molecular environment as factors contributing to the development of bone cysts (11). Given the unknown mechanism of bone cyst development, the current approach to treatment is primarily focused on managing symptoms and preventing pathological fractures (12).

However, scholars observed that when the lesion was situated near the epiphysis, complete removal was challenging to avoid harm to the epiphysis (13). Consequently, this often led to recurrence, necessitating additional surgeries. On this matter, Hunt et al. (14) reported a high recurrence rate of up to 23.8% in patients who underwent bone grafting with bone cyst scraping. Additionally, patients had to immobilize the affected limb for extended periods, limiting mobility. By 1979, Scaglietti et al. (15) introduced a treatment approach involving the local injection of methylprednisolone acetate into bone cysts. While this method was initially quick and cost-effective, it was later found to have a high recurrence rate, requiring multiple injections and negatively impacting osteogenesis. Subsequent attempts were made to overcome the osteogenic obstacles by incorporating autologous bone marrow transplantation (16), but this treatment approach proved to be less effective. Glanzmann et al. (17) reported that among 20 cases of humeral bone cysts treated with TEN, only two patients experienced recurrence at 16 and 18 months post-surgery. On the other hand, Sanctis et al. (18) conducted a prospective study on TEN, employing the Capanna score criteria. Among the 47 patients in the study with Unicameral bone cysts, 31 (65.9%) were classified as completely healed, while sixteen cases (34.1%) exhibited healing with remaining translucent areas. All these cases remained recurrence-free or non-responsive during a follow-up period of over 2 years. Furthermore, a retrospective comparative study by Wang et al. (19) investigated the necessity of TEN implantations following bone scraping and grafting for humeral bone cysts. This study found that the clinical outcomes at 16 months post-surgery were significantly better in the treatment group than in the control group (96.00% >73.91%, *P* < 0.05). The combined approach of scraped bone grafting and flexible intramedullary nailing for Unicameral bone cysts in the humerus of children was deemed to be an effective and safe treatment method without complications.

However, with the rapid advancement of new materials, there is an increasing array of options for bone grafting materials, such as allogeneic bone, autologous bone grafting, calcium sulfate bone powder, and more. In a study by Hou et al. (20), 40 patients with Unicameral bone cysts were treated using four different approaches. The method scraping of the lesion, ethanol cautery, destruction of the cyst wall, implantation of allogeneic bone, and placement of hollow screws for drainage was found to have the highest healing rate and the shortest mean healing time during short-term follow-up. In a more recent study by Li (21), the efficacy of injected calcium sulfate was compared with mixed bone grafting using allogeneic bone and autologous iliac bone. The study found no significant difference between the two groups in terms of postoperative follow-up Capanna grading for both modalities (*p* = 0.78), and both approaches demonstrated good shoulder function. The evaluation objectives of these two scholars were similar to the present study, but there were slight variations in the results. The analysis revealed that the current study evaluated all cases based on preoperative patient imaging, which accounted for approximately 9% of the total. These cases were assessed to be out of the active phase. In contrast, the two forementioned studies did not include preoperative cyst status as a reference criterion. This is noteworthy because the location of the lesion in relation to the epiphyseal plate has a significant impact on the treatment outcome (22). Furthermore, calcium sulfate injection resulted in a shorter operative time, less bleeding, and a shorter period of early partial weight-bearing on the affected limb. This can be attributed to the properties of calcium sulfate bone powder. The implantation of calcium sulfate bone powder into the medullary cavity requires only a very small puncture and fills the medullary cavity more tightly under strong pressure, which is beneficial for enhancing bone strength.

Although a minimal amount of sterile oozing has been reported in a small number of affected limbs following calcium sulfate bone powder implantation, our follow-up results indicated that the patient's puncture channel typically healed in about three days without oozing. In this study, we used allogeneic bone fillers and did not perform autologous iliac bone transplantation. This decision was the result of thorough discussions with the child and their family, who were unwilling to undergo the damage and pain associated with procedures involving other parts of the body.

In this study, the postoperative pain index for children who underwent homogeneous bone implantation was 54.5. In the case of calcium sulfate injection, it involves percutaneous puncture without the need for lesion scraping. The flexible intramedullary nails were initially inserted at multiple angles and repeatedly to penetrate the bone cyst, reduce medullary cavity pressure, and ensure the more fluid injection of calcium sulfate bone powder.

In this study, the treatment outcomes in both groups did not result in complete cures but still achieved favorable results. In the PCSI group, 7 cases (58.3%) showed complete healing of the bone cyst with no re-fractures within 24 months after surgery. The high re-operation frequency in this group may be because the timeframe for preparing the calcium sulfate artificial bone powder was relatively short. It required only a few minutes to prepare and demanded a high level of skill and coordination between the two surgeons to fill the medullary cavity adequately with artificial bone powder. One patient in this group was cured after a second operation one year later, and there was no recurrence at the last follow-up.

This study was a retrospective analysis, and as such, it carried a corresponding study bias. The number of cases in this study was relatively small, the follow-up period was short, and the long-term efficacy was not confirmed. Moreover, the young age of the children made it challenging for them to accurately describe their feelings, and all questionnaires were completed by their families. Due to complicated and various medical insurance coverage, the economic cost comparison between these two groups are unclear and the data are difficult to retrieve. In the future, we plan to continue following up with these cases and conduct prospective studies.

## 5 Conclusions

In summary, there was no significant difference in the healing rate between the two treatment modalities. This suggests that the combination of TEN with percutaneous calcium sulfate injection or LSBG is safe and effective for treating Unicameral bone cysts in the humerus. TEN combined with percutaneous calcium sulfate injection is less invasive, with shorter operative time, reduced bleeding, and less postoperative pain, although it carries the risk of multiple injections. On the other hand, TEN combined with LSBG is a more invasive approach for treating active bone cysts but offers a lower recurrence rate.

## Data Availability

The original contributions presented in the study are included in the article/Supplementary Material, further inquiries can be directed to the corresponding authors.
